# Supplementary material for: The Clinical Feature and Treatment Outcome of Ocular Melanoma: A 34-Year Experience in a Tertiary Referral Center
Source: Cancers (Basel). 2021 Nov 25;13(23):5926. doi: 10.3390/cancers13235926 (PMC8657155; doi:10.3390/cancers13235926)
Supplement: Supplementary file 1 [file cancers-13-05926-s001.zip › cancers-1454263-supplementary.pdf]

cancers

MDPI

Supplementary

Table S1: Age and gender distribution in the subgroups with melanoma of specific origins.

Tumor Origin	n	Mean Age, years (range)	*p* value
**Total**			
M	47	60.6 ± 15.9 (31–87)	0.006
F	41	50.4 ± 17.9 (12–84)	
**Uvea**			
M	26	51.7 ± 11.1 (35–72)	0.340
F	29	48.2 ± 15.8 (22–84)	
**Eyelid**			
M	4	77.3 ± 6.8 (68–84)	0.540
F	2	51.0 ± 42.4 (21–81)	
**Conjunctiva**			
M	11	64.1 ± 15.6 (31–81)	0.280
F	7	53.3 ± 22.1 (12–76)	
**Lacrimal sac**			
M	0	N/A	N/A*
F	1	57.0 (57)	
**Orbit**			
M	6	81.5 ± 4.1 (77–87)	0.430
F	2	68.5 ± 14.8 (58–79)	

*: N/A indicates that the data was not available or the study did not assess for the gene of interest.
